# NF-κB2 mutation targets survival, proliferation and differentiation pathways in the pathogenesis of plasma cell tumors

**DOI:** 10.1186/1471-2407-12-203

**Published:** 2012-05-29

**Authors:** Brian A McCarthy, Liqun Yang, Jane Ding, Mingqiang Ren, William King, Mohammed ElSalanty, Ibrahim Zakhary, Mohamed Sharawy, Hongjuan Cui, Han-Fei Ding

**Affiliations:** 1 Cancer Center and Department of Pathology, Medical College of Georgia, Georgia Health Sciences University, Augusta, GA, 30912, USA; 2 Cancer Center Genomics Core, Georgia Health Sciences University, Augusta, GA, 30912, USA; 3 Department of Oral Biology, College of Dental Medicine, Georgia Health Sciences University, Augusta, GA, 30912, USA; 4 State Key Laboratory of Silkworm Genome Biology, Institute of Sericulture and Systems Biology, College of Biotechnology, Southwest University, Chongqing, 400716, China

## Abstract

**Background:**

Abnormal NF-κB2 activation has been implicated in the pathogenesis of multiple myeloma, a cancer of plasma cells. However, a causal role for aberrant NF-κB2 signaling in the development of plasma cell tumors has not been established. Also unclear is the molecular mechanism that drives the tumorigenic process. We investigated these questions by using a transgenic mouse model with lymphocyte-targeted expression of p80HT, a lymphoma-associated NF-κB2 mutant, and human multiple myeloma cell lines.

**Methods:**

We conducted a detailed histopathological characterization of lymphomas developed in p80HT transgenic mice and microarray gene expression profiling of p80HT B cells with the goal of identifying genes that drive plasma cell tumor development. We further verified the significance of our findings in human multiple myeloma cell lines.

**Results:**

Approximately 40% of p80HT mice showed elevated levels of monoclonal immunoglobulin (M-protein) in the serum and developed plasma cell tumors. Some of these mice displayed key features of human multiple myeloma with accumulation of plasma cells in the bone marrow, osteolytic bone lesions and/or diffuse osteoporosis. Gene expression profiling of B cells from M-protein-positive p80HT mice revealed aberrant expression of genes known to be important in the pathogenesis of multiple myeloma, including cyclin D1, cyclin D2, Blimp1, survivin, IL-10 and IL-15. In vitro assays demonstrated a critical role of Stat3, a key downstream component of IL-10 signaling, in the survival of human multiple myeloma cells.

**Conclusions:**

These findings provide a mouse model for human multiple myeloma with aberrant NF-κB2 activation and suggest a molecular mechanism for NF-κB2 signaling in the pathogenesis of plasma cell tumors by coordinated regulation of plasma cell generation, proliferation and survival.

## Background

NF-κB2 is a member of the NF-κB family of transcription factors that also include NF-κB1 (p105/p50), RelA (p65), RelB, and c-Rel. The full-length NF-κB2 precursor protein p100 contains an amino-terminal Rel homology domain and a carboxyl-terminal region with seven ankyrin repeats. In response to certain cytokines, NF-κB2 is phosphorylated at specific serine residues in its carboxyl-terminal region, leading to partial proteasomal degradation of the carboxyl terminus for the production of p52. The Rel homology domain of p52 then forms active NF-κB dimers with RelB or other Rel proteins, which, once in the nucleus, bind a common DNA sequence motif known as the κB site and regulate the expression of genes crucial to the development and functions of lymphocytes [1,2].

Constitutive NF-κB2 signaling has been implicated in the pathogenesis of lymphomas. Several mechanisms have been identified wherein activation of NF-κB2 is uncoupled from its normal modes of regulation. Most of these mechanisms target upstream regulators, such as the NF-κB inducing kinase and IκB kinases [3,4]. Sustained NF-κB2 activation can also be caused by chromosomal translocations and rearrangements at the NF-κB2 locus, which occur in a variety of lymphoid malignancies including T-cell lymphoma, chronic lymphocytic leukemia, multiple myeloma, and B-cell lymphoma [5]. A cardinal feature of these genetic alterations is the generation of C-terminally truncated NF-κB2 mutants that lack various portions of the ankyrin-repeat domain [6-12]. To determine whether NF-κB2 mutation can directly initiate lymphomagenesis, we have generated transgenic mice with targeted expression in lymphocytes of p80HT, a lymphoma-associated NF-κB2 mutant [11,12]. These transgenic mice develop predominantly B-cell tumors, demonstrating that NF-κB2 mutations can have a causal role in lymphomagenesis [13].

Multiple myeloma (MM) is a common, incurable malignant tumor of plasma cells. Although much is known about individual genes and signaling pathways that are activated in MM cells, the interplay and connections between these genes and pathways that drive MM development are not well understood. Many MM cell lines have constitutively nuclear NF-κB activity and are sensitive to inhibitors of NF-κB signalling [14-16]. Recent studies have also shown that approximately 40% of MM cell lines and 17% of primary MM tumors have mutations in genes encoding regulators and effectors of NF-κB signaling, which result primarily in constitutive activation of the NF-κB2 pathway [6,17]. These findings provide genetic evidence for a critical role of NF-κB2 signaling in the pathogenesis of human MM. However, a causal relationship between aberrant activation of NF-κB2 signaling and the development of MM remains to be established. Also, it is unclear at the molecular and cellular levels how NF-κB2 signaling may drive the tumorigenic process.

In the present study, we conducted a detailed analysis of the tumors developed in p80HT mice. Our analysis revealed that approximately half of the tumors are plasma cell tumors that share some of the key pathological features of human MM. Gene expression profiling suggests that p80HT targets multiple cellular processes, including survival, proliferation and differentiation, to promote the development of plasma cell tumors.

## Methods

### Mice

p80HT transgenic mice carry the human p80HT coding sequence [11] under the control of an H-2K^b^ promoter and an immunoglobulin μ chain enhancer (pHSE3’ expression vector), which direct the transgene expression specifically in T and B lymphocytes [13,18]. The p80HT mice were generated and maintained on the C57BL/6 J x SJL/J background [13]. All animal studies were pre-approved by the Institutional Animal Care and Use Committee of Georgia Health Sciences University (GHSU).

### Histology and immunohistochemistry

Tumor samples were fixed in 10% neutral buffered formalin, embedded in paraffin, sectioned at 5 μm, and stained with Hematoxylin and Eosin (H&E). All tumor samples were examined microscopically in two independent laboratories. For immunohistochemical staining, tumor sections were deparaffinized, rehydrated, and treated with 10 mM citrate buffer (pH 6.0) at 95 °C for 25 min in water bath for antigen retrieval. After quenching of endogenous peroxidase activity with 3% H_2_O_2_ and blocking with normal rabbit serum, sections were incubated sequentially with rat anti-mouse CD138 (clone 281-2, 2.5 μg/ml, BD Pharmingen, San Diego, CA) overnight at 4 °C, biotinylated rabbit anti-rat IgG (Vector Laboratories, Burlingame, CA) 30 min at room temperature, and the ABC reagent kit (Vector Laboratories) 30 min at room temperature. The immunostaining was visualized with 3, 3′-diaminobenzidine (Sigma-Aldrich, St. Louis, MO). Section were then counterstained with Hematoxylin before being examined using a light microscope.

### Serum protein electrophoresis

Blood samples were collected into microtubes by tail bleeding and sera obtained by microfuge centrifugation for 5 min at 6,000 xg. Serum protein electrophoresis was conducted using a HYDRAGEL K-20 system according to the manufacturer’s instruction (Sebia, Norcross, GA).

### Bone analysis

For bone mineral density measurement and 3D morphometric analysis, collected femur and spine samples were scanned in a CT (computed tomography) system (Skyscan 1172, Skyscan, Aartlesaar, Belgium). Scanning was performed at an image pixel size of 36.65 μm. Reconstruction of the scanned images was done using a Skyscan Nrecon program. The reconstructed datasets loaded into Skyscan CT-analyzer software for measurement of bone mineral density and 3D morphometric parameters. Fifteen slices measuring 0.55 mm were chosen in femoral head and the body of a thoracic vertebra, and a standardized round region of interest of 1.13 mm diameter was identified along the those slices. The bone mineral density was measured in each region of interest after calibration with hydroxyl apatite phantoms of known density.

### Flow cytometry

Single-cell suspensions were prepared from the bone marrow of p80HT mice according to standard procedures. Red blood cells were lysed in ACK buffer (150 mM NH4Cl, 10 mM KHCO3, 0.1 mM EDTA, pH 7.3) and dead cells removed by passing through Lympholyte-M (Cedarlane Laboratories, Burlington, NC). The remaining cells were then stained with Phycoerythrin (PE)-conjugated rat anti-mouse CD138 (clone 281-2, 2.5 μg/ml, BD Pharmingen), sorted on a FACScan machine (BD Biosciences) and analyzed with FlowJo (Tree Star, Ashland, OR).

### Microarray

Splenic B cells were isolated from 1-year-old p80HT mice that were positive for serum M-protein and from their wild-type littermates (n = 3 per genotype) using rat anti-mouse B220 (RA3-6B2, BD Pharmingen) and magnetic beads (EasySep, Stemcell Technologies, Vancouver, BC, Canada), followed by total RNA extraction using Trizol reagent according to the manufacturer’s instructions (Invitrogen, Carlsbad, CA). RNA samples were quantified with a spectrophotometer (NanoDrop Technology, Wilmington, DE) and their quality was assessed using the Agilent 2100 Bioanalyzer (Agilent Technologies Inc., Santa Clara, CA). RNA samples were further purified with an Ambion WT Expression kit (Life Technologies, Carlsbad, CA) and then run on the Mouse Gene 1.0ST microarray chip (Affymetrix, Santa Clara, CA). Chip hybridization, washing, and imaging were performed in the GHSU Cancer Center Genomics Core using Affymetrix protocols and software with .cel and .txt files generated. Data were normalized, significance determined by ANOVA and fold change was calculated with the Partek Genomics Suite (Partek Inc., St. Louis, MO). Preliminary microarray analysis was performed by the Bioinformatics Resource in the GHSU Cancer Center Genomics Core. Further analysis was done with Microsoft Excel (Redmond, Washington), DAVID [19], and Ingenuity Pathway Analysis (IPA Ingenuity® Systems http://www.ingenuity.com), with significance of ± 1.5 fold, and P < 0.01. Depending on the software application, filters were applied to duplicates, ESTs, probes with only family similarity and hypothetical proteins. The microarray data in this manuscript are in compliance to MIAME guidelines and have been deposited in the Gene Expression Omnibus at the National Center for Biotechnology Information and is accessible through GEO Series accession number GSE30080 (http://www.ncbi.nlm.nih.gov/geo/query/acc.cgi?acc=GSE30080).

### Quantitative reverse-transcription PCR (qRT-PCR)

Relative expression of mRNA was determined on the IQ5 Detection System (Bio-Rad, Hercules, CA) using either a SYBR Green (SA Biosystems, Frederick, MD) or a TaqMan gene expression assay kit (Life Technologies), according to the manufacturer’s instructions. Independent samples (n = 3-5) were assayed twice with each assay being conducted in triplicate. TaqMan assays were performed using the following specific primers after extraction by the RNeasy Mini Kit (Qiagen, Valencia, CA): CD27 Mm01185212_g1, Ccnd1 Mm00432359_m1, Ccnd2 Mm00438071_m1, IL-15 Mm00434210_m1 and Gapdh Mm03302249_g1. The following SYBR Green primers were used after Trizol extraction: survivin forward 5′-GCGGAGGCTGGCTTCA-3′, reverse 5′-CCTGGCTCTCTGTCTGTCCA-3′; IL-10 forward 5′-TGCTATGCTGCCTGCTCTTA-3′, reverse 5′-TCATTTCCGATAAGGCTTGG-3′. Normalized mRNA expression was calculated in comparison to control samples relative to Gapdh. Significance was determined by two-tailed Student *t* test (P < 0.05).

### Cell culture

Human MM cell lines RPMI-8226 (CCL-155, ATCC, Manassas, VA) and EJM were cultured in RPMI1640 with L-glutamine, 10% FBS and 25 μg/ml gentamicin, and H929 in RPMI1640 supplemented with 10% FBS, 1% penicillin/streptomycin and 50 μM β-mercaptoethanol. All cell lines were cultured in humidified air at 37 °C and 5% CO_2_. For blocking Stat3 activity, S3I-201 (BioVision, Milpitas, CA) was dissolved in DMSO and 100 mM stock solution was prepared. The MM cell lines were treated with S3I-201 at the final concentrations of 50, 100, or 200 μM. DMSO (0.05-0.2%) was used as negative control (untreated). Trypan blue exclusion assays were performed to quantify the numbers of viable and dead cells at various time points following S3I-201 treatment.

### Immunoblotting

Cells were suspended in standard SDS sample buffer and protein concentrations were determined using a protein assay kit (Bio-Rad, Hercules, CA) with bovine serum albumin as reference. Proteins (50 μg) were separated on SDS-polyacrylamide gels, transferred to nitrocellulose membranes, and probed with the following primary antibodies: mouse anti-cyclin D1 (sc-20044, 1:200, Santa Cruz Biotechnology, Santa Cruz, CA), rabbit anti-cyclin D2 (#2924, 1:1000, Cell Signaling, Danvers, MA), rabbit anti-Stat3 (sc-482, 1:1000, Santa Cruz Biotechnology), rabbit anti-phospho-Stat3-Tyr705 (clone D3A7, #9145, 1:2000, Cell Signaling), mouse anti-survivin (clone D8, sc-17779, 1:100, Santa Cruz Biotechnology), and mouse anti-α-tubulin (B-5-1-2, 1:5000; Sigma-Aldrich). Horseradish peroxidase-conjugated goat anti-mouse and goat anti-rabbit IgG (Santa Cruz Biotechnology) were used as secondary antibodies. Proteins were visualized using a SuperSignal West Pico chemiluminescence kit (Pierce, Rockford, IL).

## Results

### p80HT mice develop B-cell and plasma cell tumors

**Figure 1:**
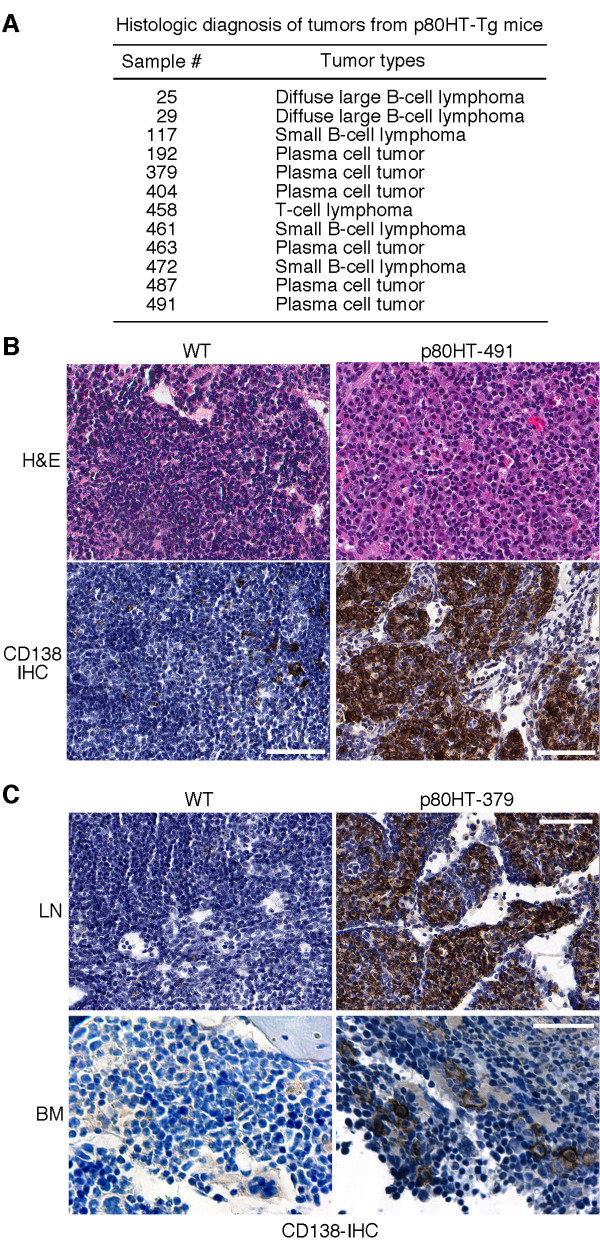
**Development of plasma cell tumors in p80HT mice.** (**A**) A summary of histological examination of tumor samples (lymph nodes) from p80HT mice. (**B**) Hematoxylin and Eosin (H&E) and CD138 immunohistochemistry staining (CD138-IHC) of the lymph node from a wild-type (WT) mouse and a representative tumor sample from the p80HT mouse #491. The tumor sample shows nodules of plasma cells expressing CD138. (**C**) CD138 immunohistochemistry staining showing accumulation of plasma cells in both the tumor (LN) and bone marrow (BM) samples from the p80HT mouse #379. Scale bars, 50 μm.

Our previous study has shown that p80HT transgenic mice develop predominantly B-cell tumors, as demonstrated by B220 immunohistochemistry staining and antigen-receptor gene rearrangements at the immunoglobulin heavy chain μ locus [13]. B-cell malignancies are a diverse group of B-cell lymphomas and leukemias, including B-lineage acute lymphoblastic leukemia, chronic lymphocytic leukemia, small lymphocytic lymphoma (also called small B-cell lymphoma), Burkitt’s lymphoma, follicular lymphoma, diffuse large B-cell lymphoma, MM, and plasmablastic lymphoma. A detailed histological examination of 12 tumor samples from the previous study [13] revealed that half of them (6/12) were plasma cell tumors, three were small B-cell lymphomas, two were diffuse large B-cell lymphomas, and one was T-cell lymphoma (Figure 1A). Immunohistochemistry staining for CD138, a marker for plasma cells, confirmed the accumulation of plasma cells in the tumors (Figure 1B and 1C for representative tumor samples). One of the three available bone marrow samples from the p80HT mice with plasma cell tumors showed an excess of bone marrow plasma cells (Figure 1C). Thus, p80HT mice develop three major types of B-lineage lymphomas.

**Figure 2:**
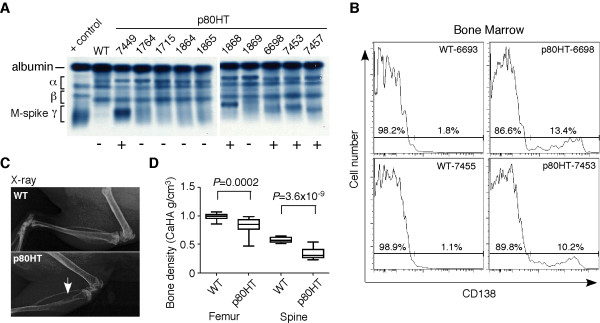
**Characterization of plasma cell tumor development in p80HT mice.**(**A**) Serum protein electrophoresis showing the presence of M-protein (+) in serum samples from p80HT mice, which is seen as a dense band or peak (M-spike) within the γ globulin region of serum proteins on an agarose gel. A pool of serum samples from patients with hypergammaglobulinemia was used as positive control. The positions of albumin and different globulin components of the serum are indicated. (**B**) Flow cytometry analysis of CD138+ plasma cell content in bone marrow samples from the #6698 and #7453 p80HT mice and their WT littermates. The bone marrow plasma cell content in both p80HT mice was more than 10%, a key criterion of MM. (**C**) X-ray on femurs and tibias from a p80HT mouse and its WT littermate. The arrow points to an area of lytic bone lesion. (**D**) Bone density analysis showing a significant reduction in bone mineral density in p80HT mice compared to age- and sex-matched WT mice (n = 5 per genotype). Data were analyzed using two-tailed Student’s *t*-test with *P* values indicated.

In light of the recent findings suggesting an important role of NF-κB2 signaling in the pathogenesis of human MM [6,17], we characterized the mouse plasma cell tumors to determine to what extent they recapitulate human MM, which is characterized by a marked increase in the bone marrow plasma cell content (>10% of the total bone marrow cellularity), monoclonal proliferation of plasma cells as indicated by the production of M-protein in the serum, osteolytic bone lesions and/or osteoporosis, and other related organ damages [20]. We collected serum samples from 25 p80HT mice at 1 year of age and serum protein electrophoresis showed that 10 of the samples (40%) contained M-protein, which was seen as a dense band or peak (M-spike) within the γ globulin region of serum proteins on an agarose gel (Figure 2A for representatives). Three of the 10 M-protein-positive p80HT mice were sacrificed for gene expression profiling (see below) and the remaining 7 mice were monitored for tumor development. Five of the 7 p80HT mice developed plasma cell tumors and became moribund within one and half years, and two of them (#6698 and #7453) had a marked increase in the bone marrow plasma cell content (>10% of the total bone marrow cellularity) compared with <2% for their WT littermates (Figure 2B). X-ray examination and bone mineral density assay revealed that bone samples from the 5 moribund p80HT mice had lytic bone lesions (Figure 2C) and a significant decrease in bone mineral density compared to the bone samples form age- and sex-matched WT littermates (Figure 2D). Together, these data demonstrate that plasma cell tumors developed in p80HT mice share key pathological features of human MM.

### Gene expression profiling of B cells from p80HT mice

**Figure 3:**
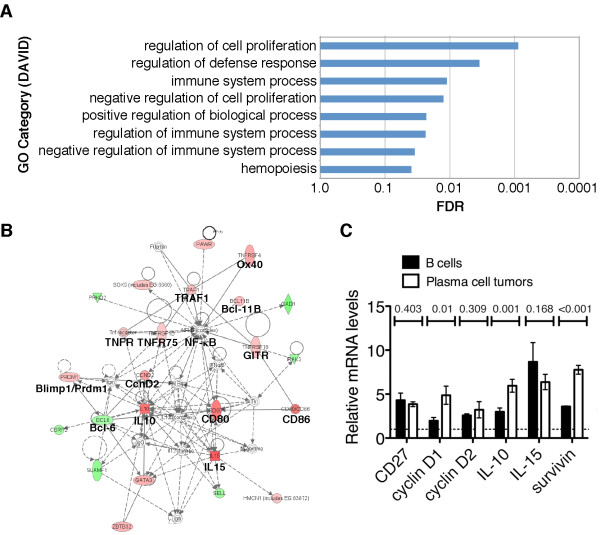
**Gene expression profiling.** (**A**) DAVID gene annotation enrichment analysis showing p80HT-regulated genes associated with various GO biological processes (FDR < 0.05). (**B**) Ingenuity pathway analysis of the same microarray data showing a network of genes relevant to human MM pathogenesis. Red indicates a significant increase in expression, green a significant decrease. Color intensity is proportional to fold change. Solid and dashed lines indicate a direct and indirect relationship, respectively. (**C**) qRT-PCR analysis of mRNA levels for CD27, cyclin D1, cyclin D2, IL-10, IL-15 and survivin in plasma cell tumor samples (n = 5) and p80HT B cells (n = 3) relative to those in wild-type B cells (n = 3, dashed line). Error bars represent the mean ± SEM of the measurements of two independent experiments with each being performed in triplicate.

To gain a molecular understanding into the oncogenic activity of p80HT in plasma cell tumor development, we examined the gene expression profile of B cells isolated from 1-year-old p80HT mice that were positive for serum M-protein and from their wild-type littermates (n = 3 per genotype). We focused on B cells based on the reasoning that identification of genes activated in these plasma cell precursors might shed light on the molecular and cellular processes that drive the development of plasma cell tumors. Microarray analysis identified a total of 201 genes that were significantly changed (± 1.5 fold, and P < 0.01), with 126 being upregulated and 75 downregulated (Additional file 1: Table S1). The top 20 up- and downregulated genes were tabulated by fold change (Table 1), and Gene Ontology (GO) analysis for biological functions revealed that many of these genes are involved in regulation of lymphocyte development, proliferation and apoptosis. We further analyzed the 201 p80HT-regulated genes by the gene annotation enrichment analysis tools DAVID and IPA. DAVID identifies the over-representation of GO terms [19], and IPA assigns biological functions to differentially expressed genes and groups them according to the biological processes in which they function (Ingenuity). The top GO biological process identified by DAVID analysis is regulation of cell proliferation (Figure 3A) and the p80HT target genes associated with the process are listed in Table 2. Many of these genes are also present in the top network identified by IPA (Figure 3B). Together, these analyses revealed a network of p80HT target genes with functional significance in the pathogenesis of MM by regulation of the differentiation, proliferation and apoptosis of plasma cells and/or MM cells.

**Table 1:** Top 20 up and downregulated genes in p80HT B cells

**Gene**	**RefSeq**	**Fold**	***P*value**	**Gene ontology/biological function**
Penk	NM_001002927	5.77	0.00098	Neuropeptide signaling pathway
Hbb-b1	NM_008220	4.87	0.00662	Positive regulation of cell proliferation
IL-15	NM_008357	4.57	0.00112	Lymph node development
Lphn2	NM_001081298	4.06	0.00248	G-protein coupled receptor protein signaling pathway
IL-10	NM_010548	4.02	0.00715	Immune response/regulation of gene expression
Hba-a2	NM_001083955	3.95	0.00213	Oxygen transport
Hba-a1	NM_008218	3.74	0.00248	Oxygen transport
B4galt6	NM_019737	3.72	0.00044	Galactosyltransferase activity
Ehf	NM_007914	3.67	0.00004	Positive regulation of transcription, DNA-dependent
CD80	NM_009855	3.65	0.00156	Positive regulation of peptidyl-tyrosine phosphorylation
Dmxl2	NM_172771	3.61	0.00185	Rab GTPase binding
Ryk	NM_013649	3.24	0.00229	Wnt receptor signaling pathway
Pmepa1	NM_022995	3.21	0.00149	Protein binding
Mllt4	NM_010806	2.95	0.00013	Cell adhesion/signal transduction
Csf2rb	NM_007780	2.88	0.00081	Cytokine-mediated signaling pathway
Ikzf2	NM_011770	2.78	0.00628	Regulation of transcription
Nt5e	NM_011851	2.77	0.00001	AMP catabolic process
Plscr1	NM_011636	2.75	0.00348	Response to virus
Tnfsf8	NM_009403	2.68	0.00839	Immune response
Ccnd2	NM_009829	2.58	0.00868	Regulation of cell cycle
Vpreb3	NM_009514	-3.95	0.00584	Pre-B cell receptor (BCR) signals
Hes1	NM_008235	-3.78	0.00235	Negative regulation of transcription
Rapgef4	NM_019688	-3.58	0.00435	Regulation of protein amino acid phosphorylation
Zfp608	NM_175751	-3.55	0.00982	Zinc ion binding
Cecr2	NM_001128151	-3.19	0.00535	Apoptosis
Tmem108	NM_178638	-3.02	0.00574	Tramsmembrane protein
CD36	NM_001159557	-2.79	0.00040	Positive regulation of cell-matrix adhesion
D8Ertd82e	NM_172911	-2.55	0.00168	Protein amino acid phosphorylation
Treml2	NM_001033405	-2.47	0.00796	T cell activation
Slc6a16	XM_914689	-2.41	0.00426	Solute carrier
Hist3h2a	NM_178218	-2.40	0.00475	Nucleosome assembly
Satb1	NM_009122	-2.36	0.00289	Negative regulation of transcription
Rftn2	NM_028713	-2.33	0.00548	Raftlin Family
Gad1	NM_008077	-2.32	0.00184	Carboxylic acid metabolic process
Gdf11	NM_010272	-2.30	0.00225	Negative regulation of cell proliferation
Itgb3	NM_016780	-2.16	0.00482	Integrin-mediated signaling pathway
Acsf2	NM_153807	-2.13	0.00050	Fatty acid metabolic process
Zfp69	NM_001005788	-2.05	0.00718	Regulation of transcription, DNA-dependent
Pip5k1b	NM_008846	-2.05	0.00710	Phosphatidylinositol metabolic process
Smad7	NM_001042660	-2.03	0.00463	Negative regulation of transcription

**Table 2:** Regulation of cell proliferation

**Gene**	**RefSeq**	**Fold**	***P*value**
IL-15	NM_008357	4.57	0.00112
IL-10	NM_010548	4.02	0.00715
CD80	BC131959	3.65	0.00156
Ccnd2	NM_009829	2.58	0.00868
Tnfrsf4	NM_011659	2.39	0.00929
Plcd3	NM_152813	2.39	0.00910
CD9	NM_007657	2.21	0.00310
Asph	NM_023066	2.16	0.00151
Adora2a	NM_009630	2.14	0.00528
Itgb1	NM_010578	2.14	0.00429
Gata3	NM_008091	2.11	0.00487
Bcl11b	NM_001079883	1.92	0.00680
CD28	NM_007642	1.90	0.00041
Prox1	NM_008937	1.89	0.00295
Pawr	NM_054056	1.79	0.00086
Fgf16	NM_030614	1.59	0.00730
Atpif1	NM_007512	1.59	0.00355
Alox8	NM_009661	1.55	0.00337
Vsig4	NM_177789	1.54	0.00016
Bcl6	NM_009744	-1.57	0.00687
Gdf11	NM_010272	-2.30	0.00225
Hes1	NM_008235	-3.78	0.00235

IL-10 (+4.02 fold, P = 0.007) promotes the proliferation and survival of MM cells [21], and cooperates with CD27 (+3.23 fold, P = 0.038) in driving the differentiation of B-cells to plasma cells [22]. IL-15 (+4.57-fold, P = 0.001) has been shown to increase proliferation and inhibit apoptosis in both MM cell lines and primary MM cells [23]. CD80 (+3.5 fold, P < 0.001) interacts with its ligand CD28 (+1.9 fold, P < 0.001) to promote the survival and proliferation of MM cells [24,25]. Blimp1/Prdm1 (+1.7 fold, P = 0.007) is a transcriptional repressor required for the formation and maintenance of mature plasma cells [26]. Upregulation of cyclin D is a major oncogenic event in MM pathogenesis [27], and in p80HT B cells, the expression of both cyclin D1 and cyclin D2 was significantly increased (cyclin D1, +2.47 fold, P = 0.038; cyclin D2, +2.58 fold, P = 0.009).

Given the well-established connection between NF-κB and TNF signaling pathways, a focus list of p80HT-regulated genes of the TNF family was independently analyzed. Nine genes with fold changes greater than ± 1.5 fold and P < 0.05 were identified (Table 3). Striking is the increase in both CD30 and CD30L. It is known that CD30L-CD30 signaling enhances cell proliferation, and an examination of a panel of B-lineage lymphomas has shown co-expression of CD30 and CD30L only in MM tumors [28].

**Table 3:** Focus list of differentially expressed TNF genes in P80HT B cells

**Gene**	**RefSeq**	**Fold**	***P*value**	**Gene ontology/biological function**
Tnfsf8/CD30L	NM_009403	2.68	0.00839	Immune response
Tnfrsf4/Txgp1	NM_011659	2.39	0.00929	Positive regulation of B cell proliferation/Ig secretion
Tnfrsf9/CD137	NM_011612	2.08	0.01446	Regulation of cell proliferation
Traf1	NM_009421	1.79	0.00461	Regulation of apoptosis
Tnfrsf8/CD30	NM_009401	1.77	0.02024	Signal transduction
Tnfrsf18/Gitr	NM_009400	1.70	0.00234	Apoptosis
Tnfrsf1b/Tnfr2	NM_011610	1.54	0.00654	Cell surface receptor linked signal transduction

We confirmed the upregulation of CD27, cyclin D1, cyclin D2, IL-10, IL-15 and survivin in p80HT B cells by qRT-PCR (Figure 3C). Survivin, also called baculoviral IAP repeat-containing protein 5 (BIRC5), is a member of the inhibitor of apoptosis proteins (IAP) and is critical for the survival of human MM cells [29]. Importantly, plasma cell tumor samples from p80HT mice (n = 5) showed even higher levels of cyclin D1, IL-10 and survivin than p80HT B cells (Figure 3C), suggesting that plasma cells or their precursor B cells with high-level expression of these genes were preferentially selected in the development of plasma cell tumors in p80HT mice.

Together, our gene expression profiling and pathway analysis suggest that p80HT promotes the development of plasma cell tumors by coordinated regulation of the generation, proliferation and survival of plasma cells.

### Stat3 inhibition induces growth arrest and cell death in human MM cell lines

**Figure 4:**
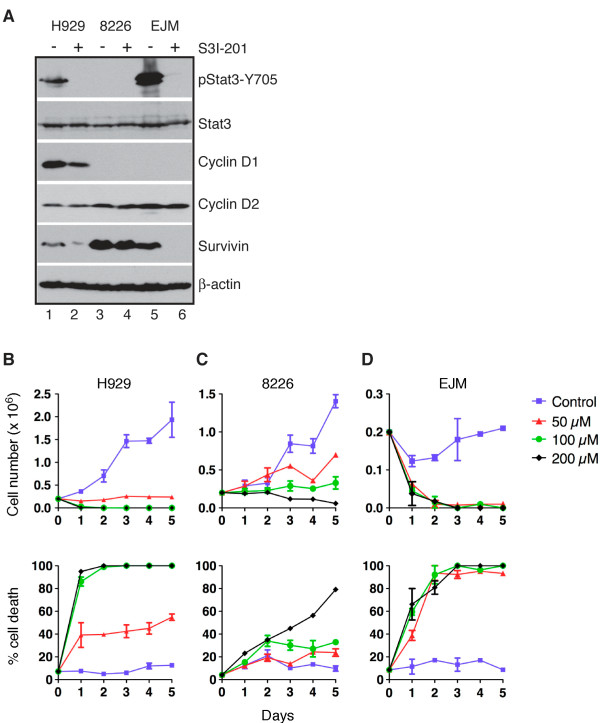
**Stat3 inhibition induces growth arrest and cell death in human MM cell lines. **(**A**) Immunoblot analysis of Stat3, pStat3-Y705, cyclin D1, cyclin D2 and survivin in human MM cell lines treated for 24 h with S3I-201 (RPMI-8226, 100 μM; H929 and EJM, 50 μM). β-actin levels are shown as loading control. (**B-D**) Quantification of cell growth and cell death of H929 (**B**), RPMI-8229 (**C**) and EJM (**D**) cells at various time points following treatment with S3I-201 at the indicated concentrations. Graphs are representative of two independent experiments.

A particularly interesting finding of above gene expression profiling and pathway analysis is the identification of a novel link between NF-κB2 and IL-10. A key component of the IL-10 signaling pathway is Stat3 [30,31], which, once activated, can transcriptionally activate a number of genes including cyclin D1, cyclin D2, survivin, and Blimp1 [32,33]. To probe the significance of this signaling pathway in the survival and proliferation of MM cells, we treated human MM cell lines with a specific inhibitor of Stat3, S3I-201, which blocks Stat3 DNA-binding and transcriptional activities by inhibiting the phosphorylation of the tyrosine residue 705 on Stat3 (pStat3-Y705) [34], an event leading to Stat3 activation. Three MM cell lines were chosen for our investigation based on their NF-κB index [16]: EJM with the highest NF-κB index, RPMI-8226 an interim index, and H929 with a low level of NF-κB activity. These cell lines were treated with S3I-201 at concentrations above and below the most commonly reported IC50 value of 100 μM [34]. Immunoblot analysis revealed no significant levels of pStat3-Y705 in RPMI-8226 cells even before S3I-201 treatment, and treatment with 100 μM S3I-201 for 24 h had no significant effect on the expression of cyclin D2 and survivin in these cells (Figure 4A, lanes 3-4). Consistent with the observation, RPMI-8226 cells were relatively resistant to S3I-201, displaying significant levels of cell death only at the concentration of 200 μM (Figure 4C). By contrast, H929 and EJM cells showed high levels of pStat3-Y705, which were markedly reduced by treatment with 50 μM S3I-201 for 24 h (Figure 4A, lanes 1-2 and 5-6). Inactivation of Stat3 in H929 and EJM cells significantly downregulated cyclin D1 and/or survivin, but had no apparent effect on cyclin D2 expression (Figure 4A, lanes 1-2 and 5-6). Consistent with the finding, both H929 and EJM cells were highly sensitive to S3I-201, with marked growth inhibition and cell death within one day of treatment with 50 μM S31-201 (Figure 4 B and 4D). EJM cells, which have higher levels of NF-κB activity [16] and pStat3-Y705 than H929 cells (Figure 4A), were also more susceptible to S3I-201: approximately 90% of EJM cells lost viability in the presence of 50 μM S31-201 within 2 days (Figure 4C), whereas H929 cells showed only 40% of cell death under the same condition (Figure 4D). Thus, in MM cells with high levels of Stat3 activation (pStat3-Y705), the Stat3 signaling is crucial for the expression of cyclin D1 and survivin, and the proliferation and survival of cells.

## Discussion

Despite much effort, the development of mouse models for human MM remains a challenge [35]. A major advance in this area of research has been provided recently by the generation of a transgenic mouse line with spontaneous MYC activation driven by Activation-Induced Deaminase (AID) [36]. These transgenic mice developed bone marrow plasma cell tumors that recapitulate many features of human MM. However, rearrangements of the MYC gene are present in only 15% of MM patients [37], calling for the development of additional mouse models that target distinct signaling pathways important in MM pathogenesis. Chromosomal translocations and rearrangements at the NF-κB2 locus have been shown to occur in primary human MM [8]. The human MM cell lines JK6L and CAG also carry genetic mutations in the NF-κB2 gene [6]. These mutations lead to the generation of C-terminally truncated NF-κB2 proteins similar to the tumor-derived NF-κB2 mutant p80HT [11]. Currently, no mouse models are available for human MM with aberrant activation of NF-κB2 signaling. We have recently reported that transgenic mice with targeted expression of p80HT in lymphocytes developed predominantly B-lineage lymphomas with the tumor incidence of 79% by 70 weeks [13]. In this investigation, we conducted detailed histological and immunohistochemistry examination of 12 tumor samples from the previous study [13], which revealed that half of them (6/12) were plasma cell tumors. To corroborate this finding, we generated additional p80HT mice with a focus on their development of plasma cell tumors. Approximately 40% of the newly generated p80HT mice produced M-protein by 1 year of age. Most of these M-protein-positive p80HT mice developed plasma cell tumors with diffuse osteoporosis. Some of them also had osteolytic bone lesions and/or significant accumulation of plasma cells in the bone marrow. These findings provide the first direct evidence for a causal role of NF-κB2 mutation in the pathogenesis of plasma cell tumors that share some key histopathological and clinical features of human MM.

The transgenic mice express p80HT in both T and B cells, and our previous study suggests that p80HT promotes tumor development primarily by enhancing the survival of T and B cells [13]. Also, the lymphoma development in p80HT mice is characterized by a prolonged latent period [13], suggesting that additional genetic and/or epigenetic alterations are required for the malignant transformation of p80HT lymphocytes and their clonal expansion. We speculate that this might be the major reason why p80HT mice develop a wide spectrum of B cell lymphomas including plasma cell tumors, as well as T cell lymphomas, which depend on the type and developmental stage of the lymphocytes that have acquired secondary genetic and/or epigenetic alterations.

We have previously identified TRAF1 as a target gene critical for the oncogenic activity of p80HT [13]. TRAF1 deficiency reestablished B cell homeostasis and significantly delayed the tumor development in p80HT mice [13] (unpublished data). However, constitutive overexpression of TRAF1 in lymphocytes is not tumorigenic in mice [38] suggesting that additional target genes must be critical for the development of plasma cell tumors in p80HT mice. We performed gene expression profiling of B cells from M-protein-positive p80HT mice for the reason that genes activated in these plasma cell precursors are anticipated to drive the development of plasma cell tumors. It is important to note that the three M-protein-positive p80HT mice used for the microarray assay might not develop plasma cell tumors in the end. Nonetheless, the gene expression profiling revealed the activation of many genes, in addition to TRAF1, in p80HT B cells that are known to promote the proliferation and survival of MM cells, as well as the differentiation of B cells to plasma cells.

IL-15 is the second most upregulated gene in p80HT B cells. IL-15 is important to the proliferation of MM cells and their ability to evade apoptosis [23]. An autocrine loop between IL-15 and its receptor has been identified as a mechanism for tumor cell expansion in MM [39]. The upregulation of both IL-15 and its receptor IL-15Ra (+1.7 fold, P = 0.045) in the B cells of p80HT mice suggest that this pathway is important in the pathogenesis of plasma cell tumors in our mouse model.

Another top upregulated gene is IL-10. Using the transcription factor database search tool DECODE (SaBiosciences) and data from the University of California Santa Cruz Genome Browser, we identified potential two κB-binding sites in the IL-10 promoter (unpublished data), suggesting that p80HT may directly upregulate IL-10 expression. IL-10 exerts its biological functions primarily through Stat3 [30,31]. It has long been recognized that IL-10 promotes the differentiation of B cells to plasma cells [22,40-43]. This action of IL-10 is likely mediated, at least in part, by Blimp1, a known target gene of Stat3 [33]. Consistent with the notion, Blimp1 expression was significantly upregulated (+1.7 fold, P = 0.007) in p80HT B cells. Blimp1 is a transcriptional repressor required for the formation and maintenance of mature plasma cells [26]. Blimp1 is also required for the formation of plasmacytoma in a mouse model [44]. Other Stat3 target genes include cyclin D1, cyclin D2, and survivin [32,33], and they were all markedly upregulated in p80HT B cells.

Survivin is a member of the inhibitor of apoptosis protein family with dual roles in regulation of cell cycle progression and apoptosis [45]. Survivin expression is increased during MM progression, and knockdown of survivin induces cell death in human MM cells [29]. It has been shown previously that the human survivin gene promoter region contains κB-binding sites [46], and our sequence examination revealed 4 potential κB-binding sites within the mouse survivin promoter region (unpublished data). These observations suggest that p80HT may transcriptionally upregulate survivin expression either directly or indirectly through the IL-10-Stat3 signaling pathway.

Cyclins D1, D2 and D3 (encoded by CCND1, D2 and D3) interact with and activate cyclin-dependent kinase 4 (Cdk4) or Cdk6 to facilitate the G1/S cell-cycle transition [47]. Upregulation of cyclin D expression occurs in the vast majority of MM tumors and has been considered a crucial and early oncogenic event in MM pathogenesis [27]. Approximately 20% of MM tumors show elevated levels of cyclin D1 or D3 as the result of chromosomal translocations that juxtapose potent immunoglobulin (Ig) gene enhancers next to CCND1 (11q13) or CCND3 (6p21) [27], and ~7% of MM tumors have Ig translocations involving c-MAF (16q23) or MAFB (20q11), which encode transcription factors that target CCND2 [27,48]. In the rest of MM tumors, the transcription factors responsible for upregulating the expression of cyclin D genes remain to be identified. Cyclin D1 is a known target gene of the NF-κB1 signaling pathway [49,50]. However, to the best of our knowledge, a role for NF-κB2 in regulation of cyclin D expression has not been previously described. Thus, our findings that cyclin D1 and cyclin D2 were significantly upregulated in p80HT B cells suggest a novel mechanism for early activation of cyclin D genes in the development of MM.

**Figure 5:**
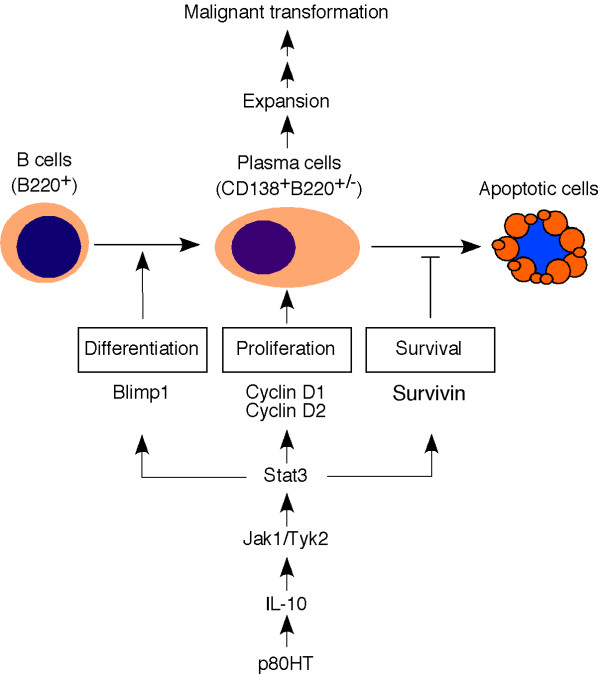
A simplified model for p80HT targeting the IL-10-Stat3 signaling pathway to promote plasma cell expansion and tumor development.

On the basis of above discussion, we suggest a model for p80HT activation of the IL-10-Stat3 signaling pathway to promote plasma cell expansion and tumor development by regulating the generation, proliferation and survival of plasma cells (Figure 5). Please note that p80HT may also directly activate the transcription of cyclin D1, cyclin D2 and survivin genes. In addition, other p80HT-regulated genes, including IL-15, CD27, CD28, CD30, CD30L, CD80, and TRAF1 may contribute significantly to the tumorigenic process.

To determine the clinical relevance of our findings, we assessed the response of human MM cell lines to the inhibition of Stat3, a key downstream component of the IL-10 signaling pathway. Our investigation revealed a correlation between the activity of Stat3 and the sensitivity to Stat3 inhibition in MM cells. Also consistent with our model, Stat3 signaling was found to be essential for high-level expression of cyclin D1 and survivin in MM cells, providing a molecular mechanism for the critical role of Stat3 signaling in the proliferation and survival of MM cells. Thus, targeting the Stat3 signaling pathway may represent a therapeutic strategy for human MM.

## Conclusions

Our findings provide the first direct evidence for a causal role of NF-κB2 mutation in the pathogenesis of mouse plasma cell tumors that share some key histopathological and clinical features of human MM. The NF-κB2 mutant p80HT promotes the development of plasma cell tumors by transcriptional activation of genes critical for the generation, proliferation and survival of plasma cells, including cyclin D1, cyclin D2, Blimp1, survivin, IL-10 and IL-15. We further present evidence that targeting the IL-10-Stat3 signaling pathway may represent a therapeutic strategy for human MM.

## Competing interests

The authors declare that they have no competing interests.

## Authors’ contributions

Conceived and designed the experiments: BAM LY HC HFD. Performed the experiments: BAM LY JD MR WK ME IZ. Analyzed the data: BAM LY JD MR WK ME IZ MS HFD. Wrote the paper: BAM LY HC HFD. All authors read and approved the final manuscript.

## Pre-publication history

The pre-publication history for this paper can be accessed here:

http://www.biomedcentral.com/1471-2407/12/203/prepub

## Supplementary Material

Additional 1: Table S1. Genes significantly altered in p80HT B cells with Gene Ontology (GO) annotations. Click here for file
